# Supplementary material for: Meningeal lymphatic supporting cells govern the formation and maintenance of zebrafish mural lymphatic endothelial cells
Source: Nat Commun. 2024 Jul 2;15:5547. doi: 10.1038/s41467-024-49818-5 (PMC11220022; doi:10.1038/s41467-024-49818-5)
Supplement: Supplementary file 1 — Supplementary Information [file 41467_2024_49818_MOESM1_ESM.pdf]

Supplementary information for

Meningeal lymphatic supporting cells govern the formation and maintenance of zebrafish mural lymphatic endothelial cells

Xiang He[1,4], Daiqin Xiong[1,4], Lei Zhao[2], Jialong Fu[1], and Lingfei Luo[1,3,*]

[1]Institute of Developmental Biology and Regenerative Medicine, Southwest University, Beibei 400715, Chongqing, China

[2]Shaanxi Key Laboratory of Qinling Ecological Intelligent Monitoring and Protection, School of Ecology and Environment, Northwestern Polytechnical University, Xi'an 710072, Shaanxi, China

[3]School of Life Sciences, Fudan University, Yangpu 200438, Shanghai, China

[4]These authors contributed equally

***Correspondence**: lluo@swu.edu.cn

Supplementary Figures and Legends

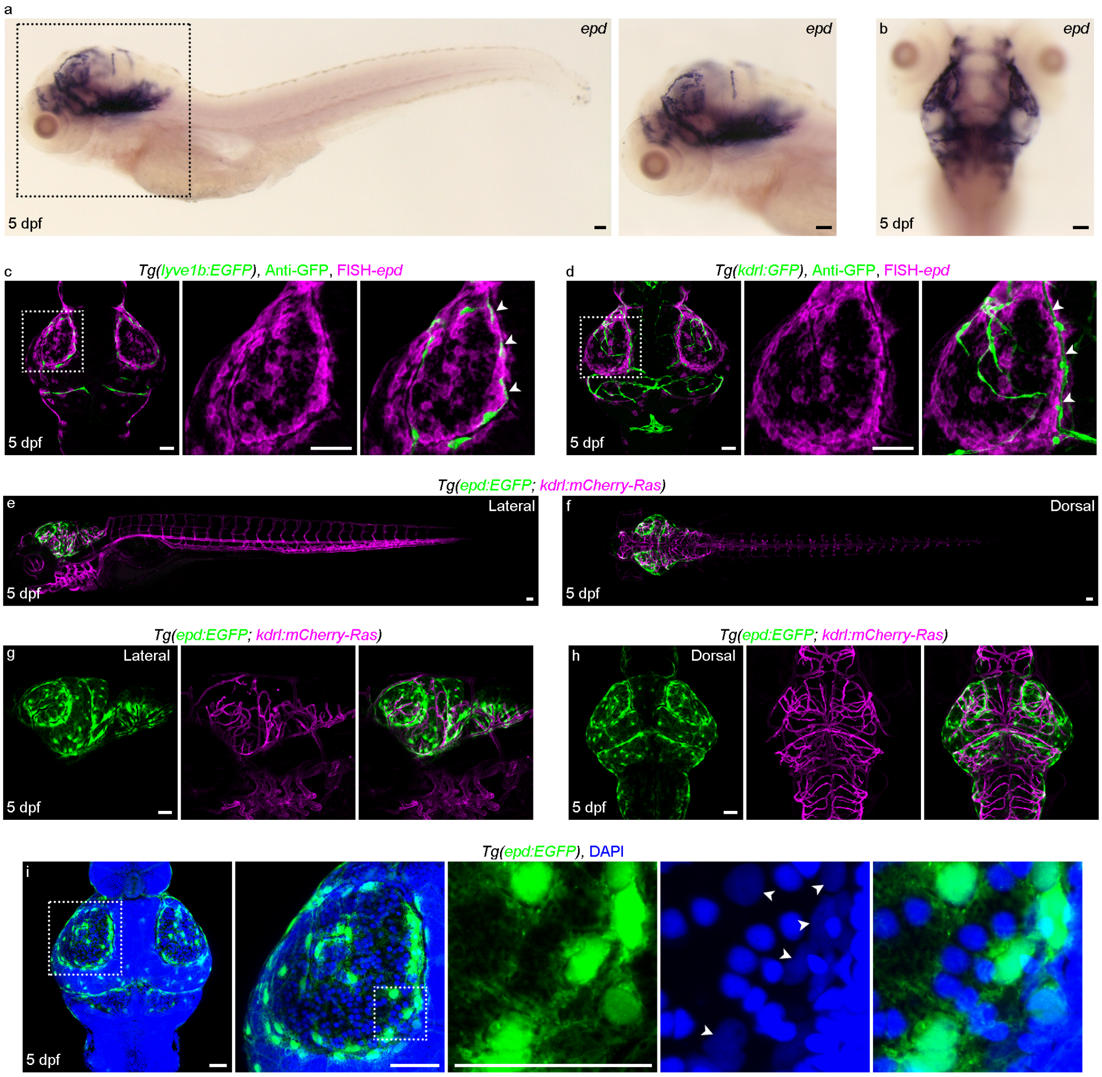

Supplementary Fig. 1 | The *epd*-positive cells are present exclusively in the zebrafish brain. a, **b** Whole-mount in situ hybridization (WISH) of *epd* in wild-type larvae at 5 dpf. Lateral (**a**, *n* = 30) and dorsal (**b**, *n* = 30) views of the frame region are shown. The experiment was repeated three times independently with similar results. **c**, **d** Dorsal confocal images of fluorescence in situ hybridization (FISH) combined with antibody staining (FISH-antibody staining) of *epd* in

Tg(lyve1b:EGFP) (**c**, *n* = 15) and *Tg(kdrl:GFP)* (**d**, *n* = 15) larval brains at 5 dpf. White arrowheads indicate the mesencephalic vein (MsV). Each experiment was repeated three times independently with similar results. **e**, **f** Representative images of the presence of *epd*-positive cells in *Tg(epd:EGFP; kdrl:mCherry-Ras)* larval brains at 5 dpf. Lateral (**e**) and dorsal (**f**) views of the whole larval zebrafish are shown. *n* = 20 per panel. **g**, **h** Representative images of the distributions of *epd*-positive cells in larval brains at 5 dpf. Lateral (**g**, *n* = 25) and dorsal (**h**, *n* = 25) views of the larval zebrafish brains are shown. **i** Dorsal confocal images of DAPI staining of *Tg(epd:EGFP)* showed *epd*-positive cell nuclei at 5 dpf. White arrowheads indicate DAPI labelled *epd*-positive cell nuclei. *n* = 20. The black and white dashed boxes outline the enlarged areas. Scale bars: 50 μm.

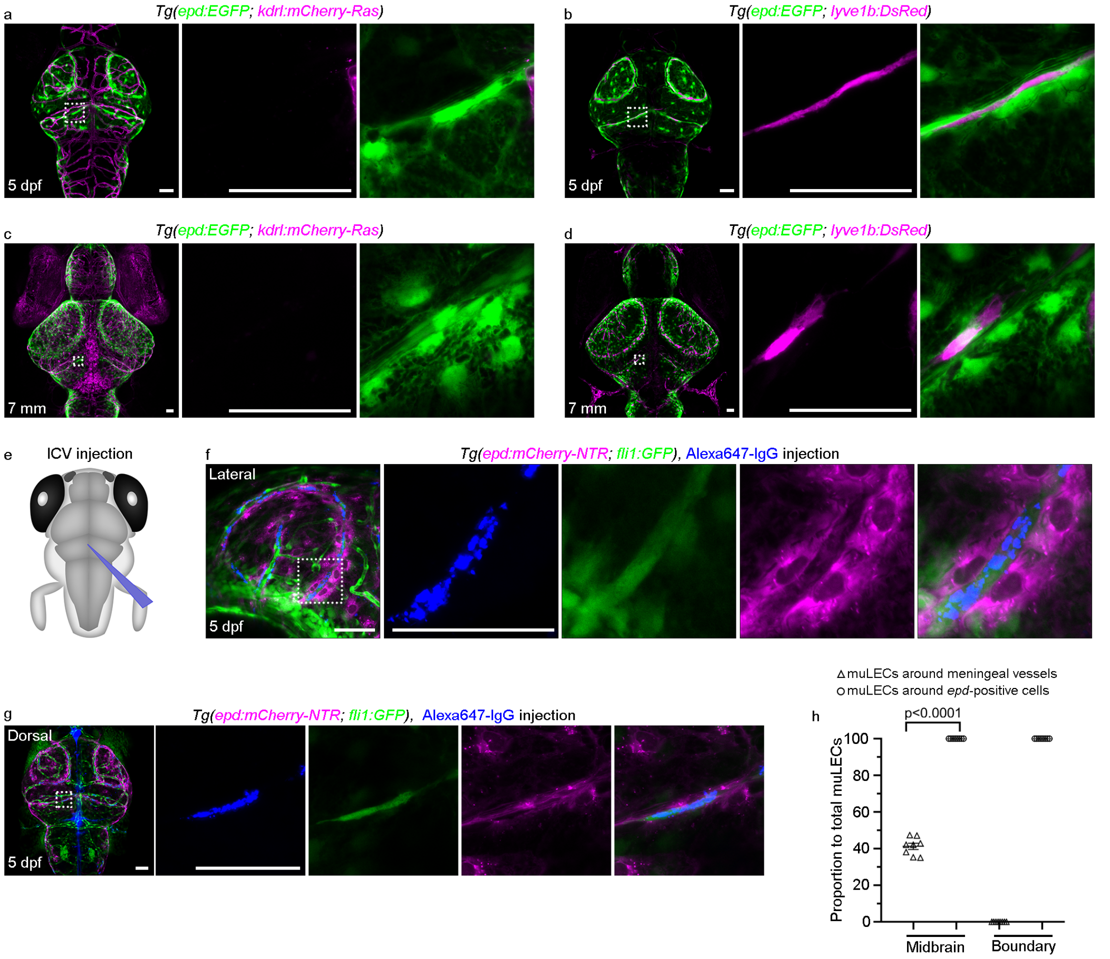

Supplementary Fig. 2 | No blood vessels but *epd*-positive cells present around muLECs in some meningeal areas of larval and juvenile zebrafish. a, **c** Dorsal confocal images of *kdrl:mCherry-Ras*-positive blood vessels and *epd*-positive cells on *Tg(epd:EGFP; kdrl:mCherry-Ras)* brains at 5 dpf (**a**, *n* = 20) and 7 mm (**c**, *n* = 20). The magnified boxed areas showed the absence of meningeal blood vessels and the presence of *epd*-positive cells. **b**, **d** Dorsal confocal images of *lyve1b:DsRed*-positive mural lymphatic endothelial cells (muLECs) and *epd*-positive cells on *Tg(epd:EGFP; lyve1b:DsRed)* brains at 5 dpf (**b**, *n* = 20) and 7 mm (**d**, *n* = 20). The enlarged boxed areas showed the presence of muLECs and

epd-positive cells. **e** Illustration of intracerebroventricular (ICV) injection point of Alexa647-IgG. **f**, **g** Confocal images of the absorption of Alexa647-IgG by muLECs in larval brains. The magnified boxed areas showed presence of muLECs and *epd*-positive cells as well as absence of meningeal blood vessels in the posterior half of the midbrain (**f**, *n* = 15) and the midbrain-hindbrain junction (**g**, *n* = 15). Lateral (**f**) and dorsal (**g**) views of the larval brains are shown. The experiment was repeated three times independently with similar results. **h** The statistic graph showed the number of muLECs around meningeal vessels and the number of muLECs around *epd*-positive cells as a proportion of the total number of muLECs, respectively, over the midbrain and the midbrain-hindbrain boundary at 5 dpf. 8 fish were observed in three independent experiments in each group. Error bars, mean ± SEM. Unpaired two-tailed Student's *t*-test. *P* value included in the graph. Source data are provided as a Source Data file. The white dashed boxes outline the enlarged areas. Scale bars: 50 μm.

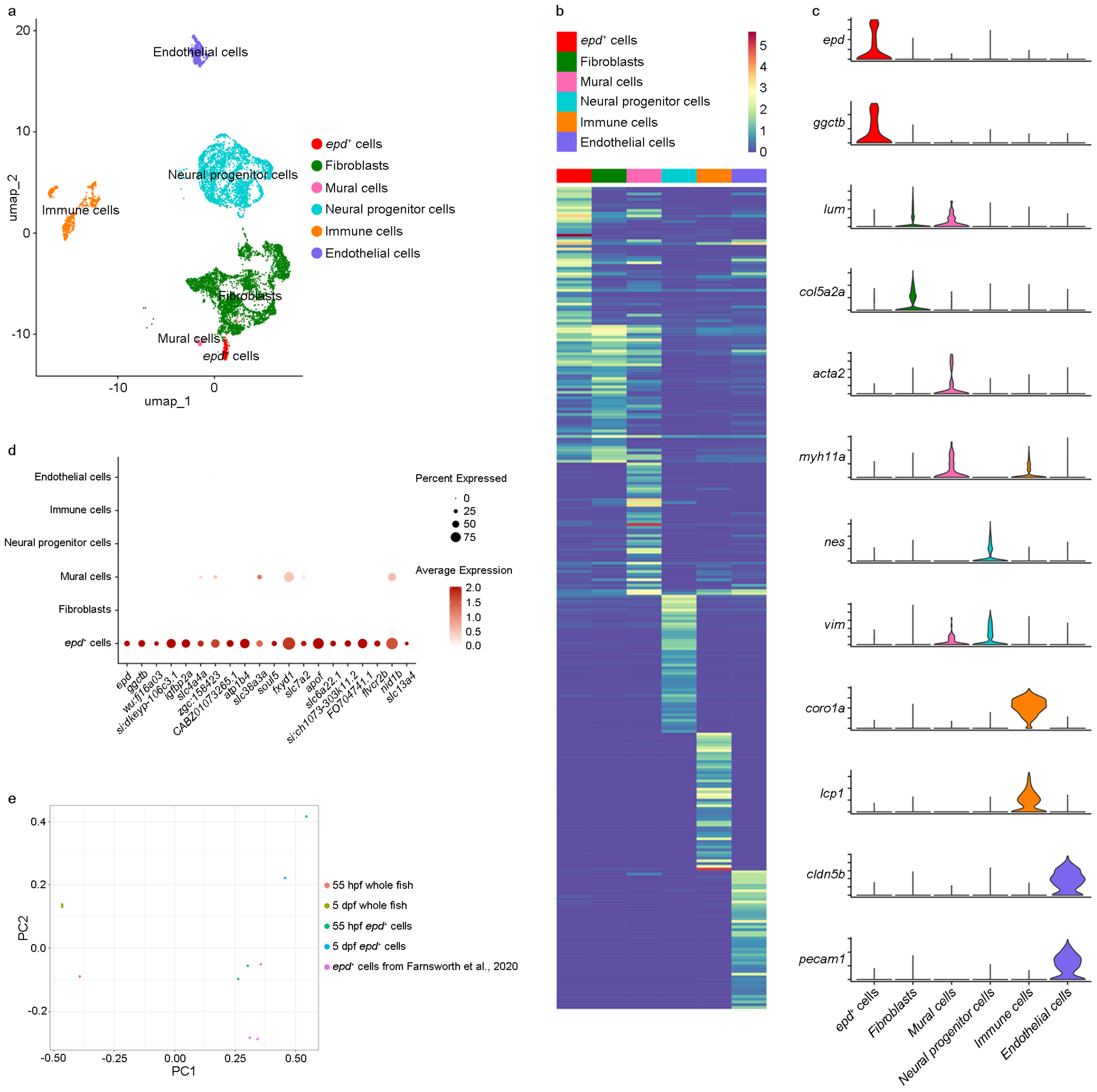

Supplementary Fig. 3 | The *epd*-positive cells differ from other meningeal cell types from the single cell transcriptome level. a A Uniform Manifold Approximation and Projection (UMAP) plot of selected cell clusters mined from Farnsworth et al., 2020. Cell clusters were distinguished by different colors and annotated according to their transcriptional characteristics. **b** Heatmap of the top 50 marker genes across all cell clusters. **c** The violin plot showing the expression of representative marker genes for all cell types. **d** The dotplot showing the

expression of top 20 marker genes (horizontal axis) for *epd*-positive cells in each cell cluster (vertical axis). Dot size indicates the percentage of cells that express the marker. Dot color indicates the average ratio expression level of cells expressing the marker. **e** Principle component (PC) analysis comparing RNA sequencing data of whole fish at 55 hpf and 5 dpf, *epd*-positive cells at 55 hpf and 5 dpf, and *epd*-positive cells mined from Farnsworth et al., 2020.

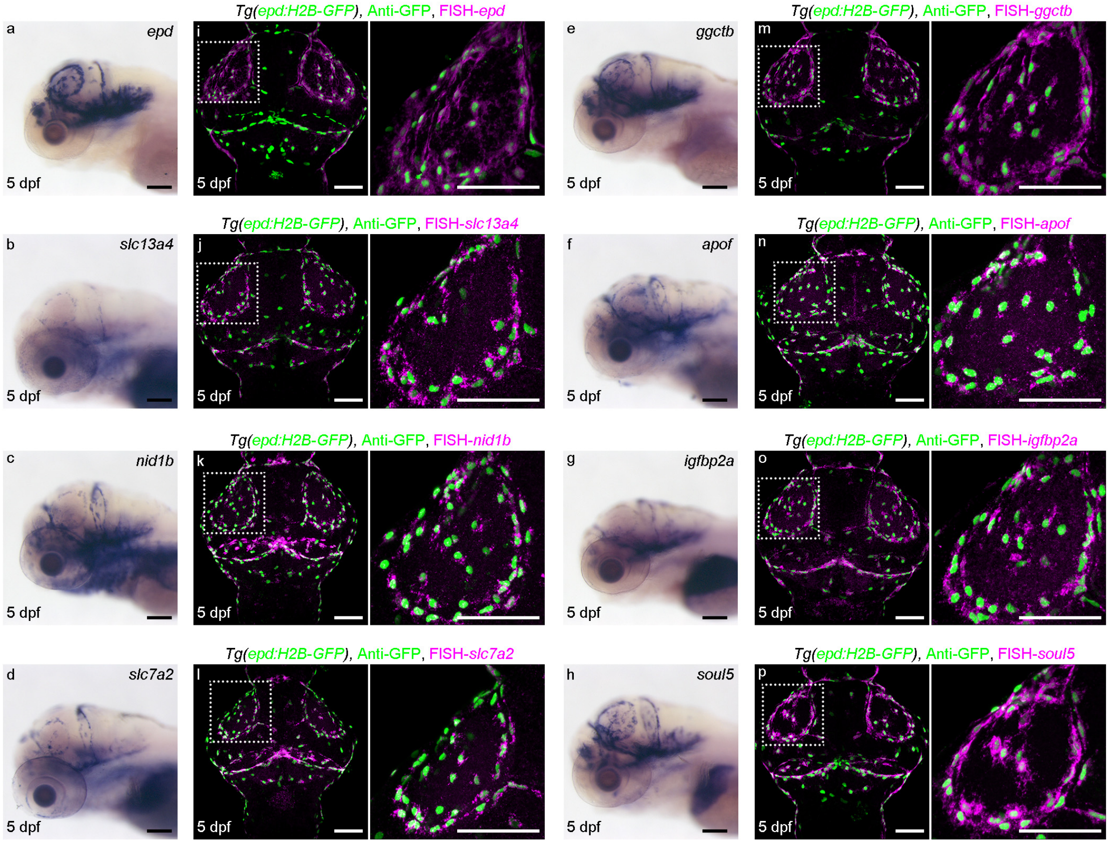

Supplementary Fig. 4 | The *epd*-positive cells express exclusive markers. a-h Lateral images of whole-mount in situ hybridization (WISH) of *epd*-positive cell marker genes *epd* (*n* = 20), *slc13a4* (*n* = 18), *nid1b* (*n* = 17), *slc7a2* (*n* = 17), *ggctb* (*n* = 18), *apof* (*n* = 18), *igfbp2a* (*n* = 17), and *soul5* (*n* = 16) in wild-type zebrafish larvae at 5 dpf. Each experiment was repeated three times independently with similar results. **i-p** Dorsal confocal images of fluorescence in situ hybridization (FISH)-antibody staining of *epd*-positive cell marker genes *epd* (*n* = 15), *slc13a4* (*n* = 14), *nid1b* (*n* = 13), *slc7a2* (*n* = 14), *ggctb* (*n* = 13), *apof* (*n* = 13), *igfbp2a* (*n* = 14) and *soul5* (*n* = 13) in wild-type *Tg(epd:H2B-GFP)* brains at 5 dpf. Each

experiment was repeated three times independently with similar results. The white dashed boxes outline the enlarged areas. Scale bars: 100 μm.

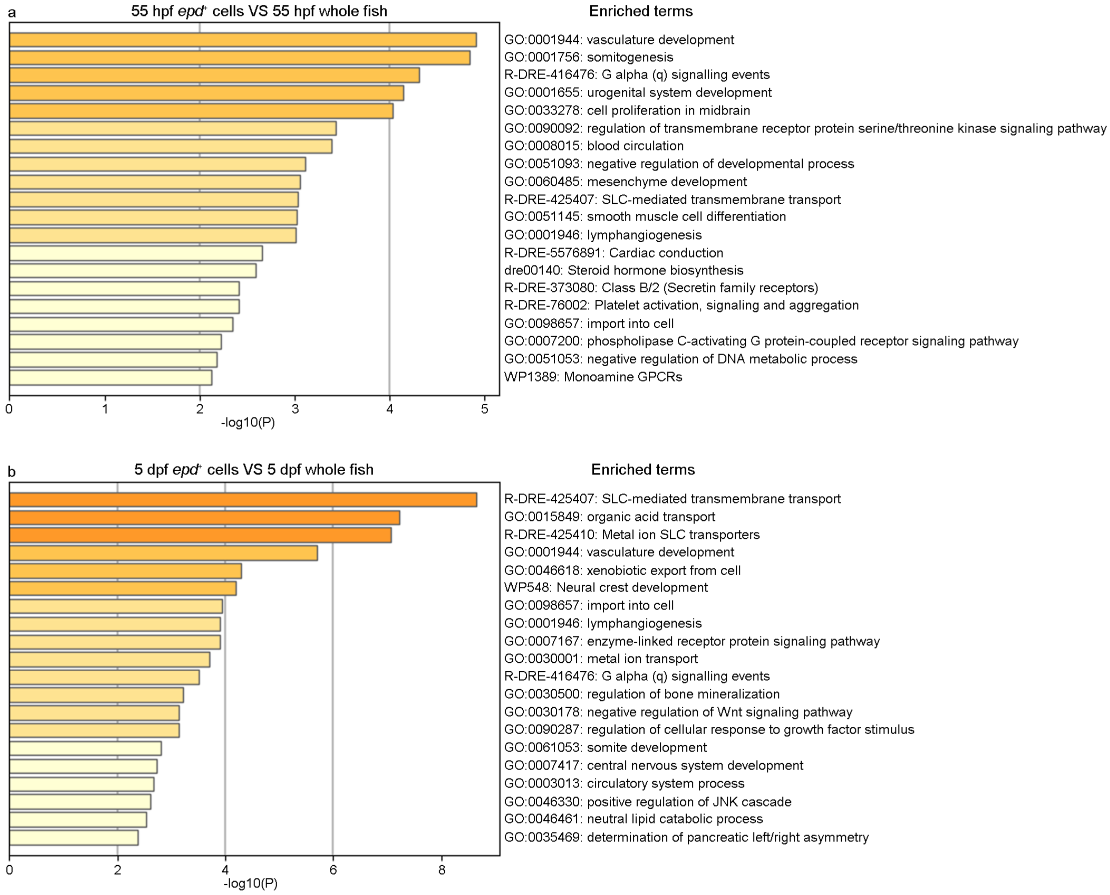

Supplementary Fig. 5 | Gene enrichment ontology analysis of differentially expressed genes between *epd*-positive cells and whole fish at embryonic/larval stages. a, **b** Gene enrichment ontology analysis, including gene ontology (GO), kyoto encyclopedia of genes and genomes (KEGG) terms, canonical pathways, and hall mark gene, showed significant enriched terms for highly expressed genes in *epd*-positive cells relative to whole fish at 55 hpf (**a**) and 5 dpf (**b**).

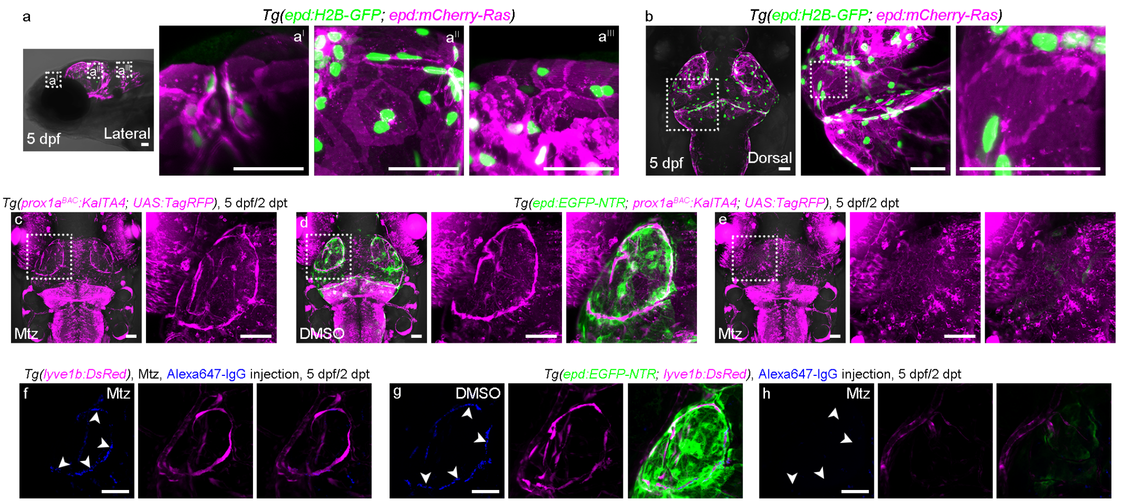

Supplementary Fig. 6 | Ablation of *epd*-positive cells results in defective development of muLECs. a, **b** Confocal images of single cell morphology of *epd*-positive cells in *Tg(epd:H2B-GFP; epd:mCherry-Ras)* larvae at 5 dpf. **a**$^{\mathbf{I}}$, forebrain. **a**$^{\mathbf{II}}$, midbrain. **a**$^{\mathbf{III}}$, hindbrain. Lateral (**a**) and dorsal (**b**) views of the larval brains were shown. *n* = 20 per experiment. **c**, **d** Dorsal confocal images of mural lymphatic endothelial cells (muLECs) and *epd*-positive cells in Mtz-treated *Tg(prox1a*^{BAC}*:KalTA4; UAS:TagRFP)* brains (**c**, *n* = 37) and DMSO-treated *Tg(epd:EGFP-NTR; prox1a*^{BAC}*:KalTA4; UAS:TagRFP)* brains (**d**, *n* = 35) at 5 dpf/2 dpt. **e** Dorsal confocal images of the absence of muLECs in Mtz-treated *Tg(epd:EGFP-NTR; prox1a*^{BAC}*:KalTA4; UAS:TagRFP)* brains at 5 dpf/2 dpt. *n* = 33. The experiment was repeated three times independently with similar results. **f**, **g** Dorsal confocal images of the absorption of Alexa647-IgG by muLECs in Mtz-treated *Tg(lyve1b:DsRed)* brains (**f**, *n* = 12) and DMSO-treated *Tg(epd:EGFP-NTR; lyve1b:DsRed)* brains (**g**, *n* = 10) at 5 dpf/2 dpt. Each experiment was repeated

three times independently with similar results. Arrowheads indicate Alexa647-IgG taken up by muLECs. **h** Dorsal confocal images of the absent absorption of Alexa647-IgG in muLEC areas in Mtz-treated *Tg(epd:EGFP-NTR; lyve1b:DsRed)* brains at 5 dpf/2 dpt. The experiment was repeated three times independently with similar results. White arrowheads indicate where muLECs should have been located. *n* = 9. The white dashed boxes outline the enlarged areas. Scale bars: 50 μm.

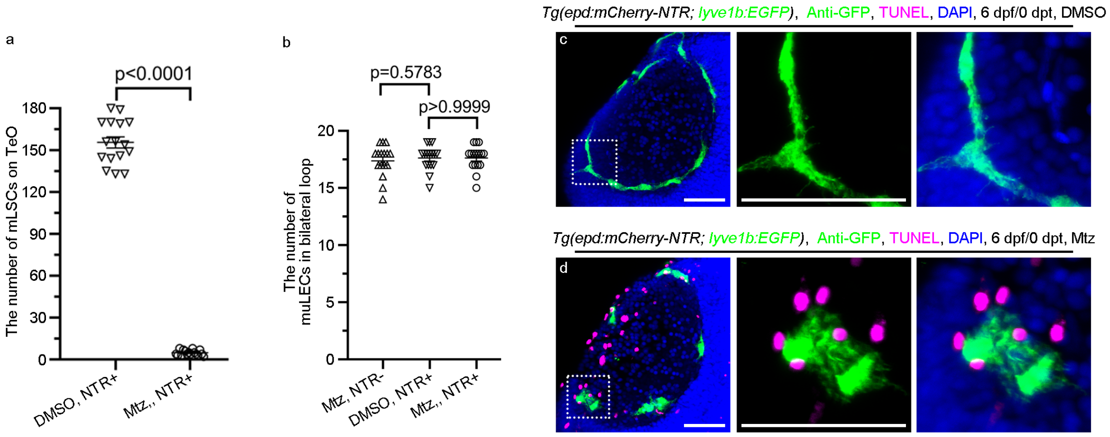

Supplementary Fig. 7 | The mLSC ablation does not lead to muLEC reduction and apoptosis in the larval stage. a, **b** Quantification of the number of meningeal lymphatic supporting cells (mLSCs, equivalent to *epd*-positive cells) on the optic tectum (TeO) at 6 dpf/0 dpt (**a**) and the number of mural lymphatic endothelial cells (muLECs) in bilateral loop at 6 dpf/0 dpt (**b**) in the nonablation and mLSC ablation groups. 16 fish were observed in three independent experiments in each group. **c**, **d** Dorsal confocal images of muLECs and TUNEL signals on the *Tg(epd:mCherry-NTR; lyve1b:EGFP)* larval brains at 6 dpf/0 dpt. Each experiment was repeated three times independently with similar results. Error bars, mean ± SEM. Unpaired two-tailed Student's *t*-test. *P* values included in the graphs. Source data are provided as a Source Data file. The white dashed boxes outline the enlarged areas. Scale bars: 50 μm.

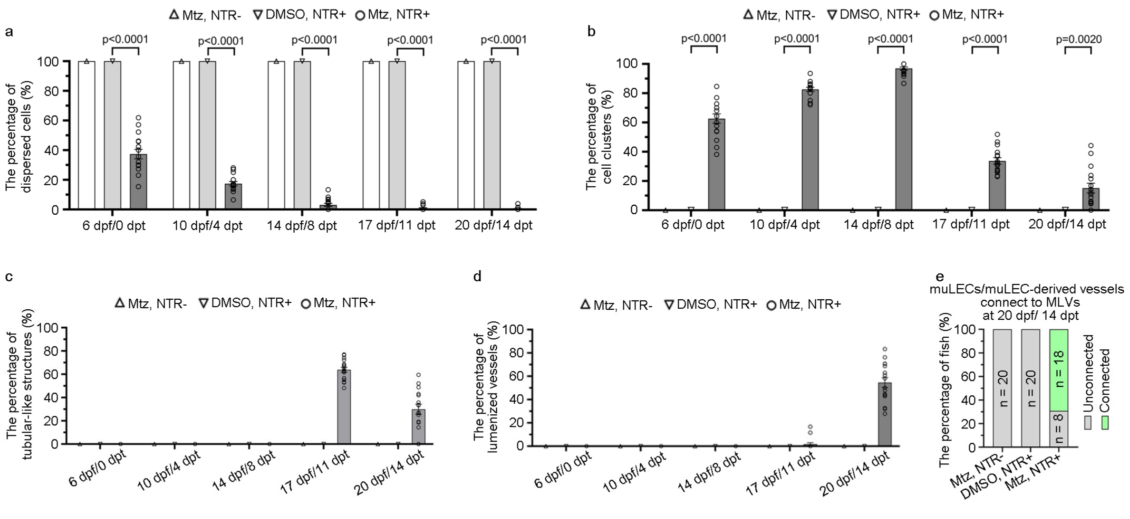

Supplementary Fig. 8 | Quantification of the morphological maintaining role of mLSCs on muLECs. a-d Quantification of the percentage of the number of mural lymphatic endothelial cells (muLECs) in the form of dispersed cells (**a**), cell clusters (**b**), tubular-like structures (**c**) and lumenized vessels (**d**) to the total muLECs on the dorsal of the brain in the nonablation and meningeal lymphatic supporting cell (mLSC) ablation groups, respectively, at different time points. 16 fish were observed in three independent experiments in each group. The same larvae were used for the same period. **e** Percentage of muLECs and muLEC-derived vessels connected (green) and unconnected (gray) to meningeal lymphatic vessels (MLVs) at 20 dpf/14 dpt. Mtz, NTR-*,* *n* = 20; DMSO, NTR+, *n* = 20; Mtz, NTR+, *n* = 26. Each experiment was repeated three times independently with similar results. Error bars, mean ± SEM. Two-way ANOVA Sidak's multiple comparisons test. *P* values included in the graphs (**a-d**). Source data are provided as a Source Data file.

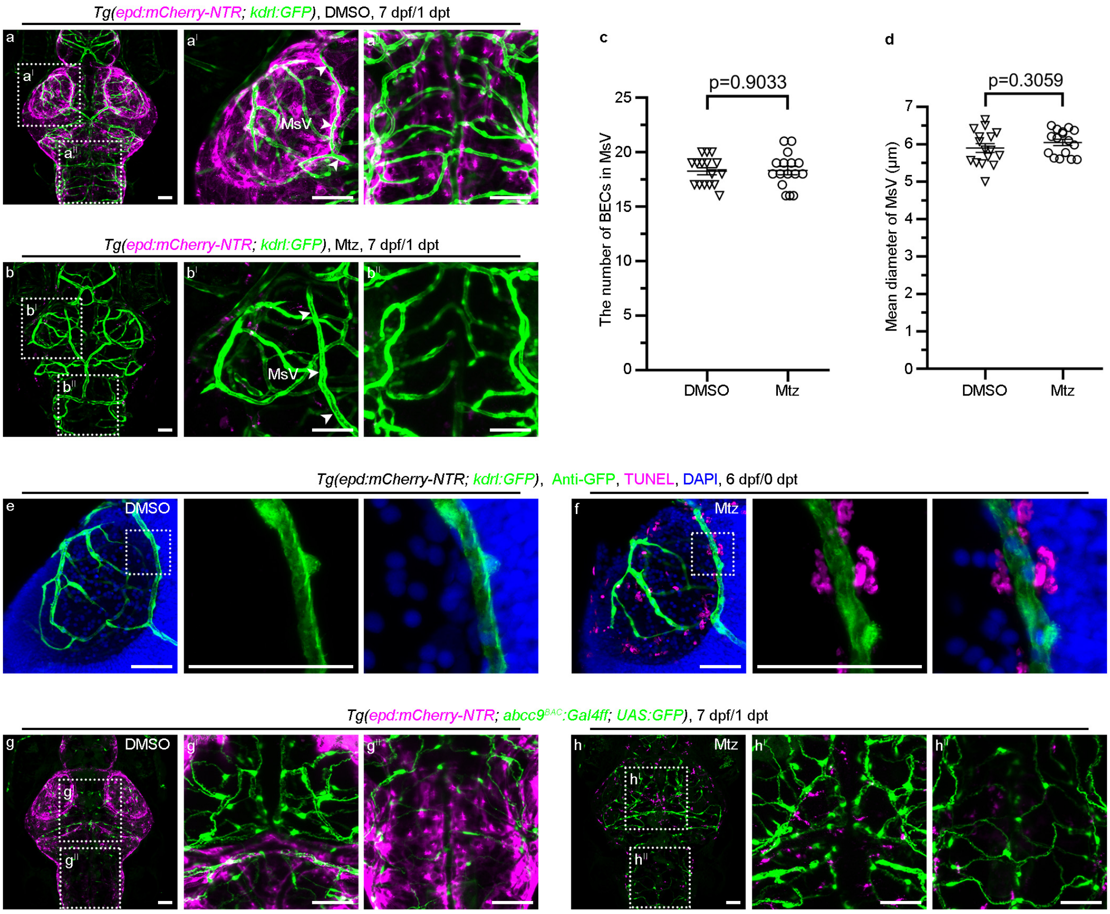

Supplementary Fig. 9 | Ablation of mLSCs does not cause BEC apoptosis and pericyte changes. a, **b** Dorsal confocal images of meningeal lymphatic supporting cells (mLSCs) and blood vessels on *Tg(epd:mCherry-NTR; kdrl:GFP)* larval brains at 7 dpf/1 dpt. **a^{I}**, **b^{I}** showed the left midbrain. **a^{II}**, **b^{II}** showed the hindbrain. **a**, *n* = 43; **b**, *n* = 37. Each experiment was repeated three times independently with similar results. **c**, **d** Quantification of the number of blood vessel endothelial cells (BECs) in the mesencephalic vein (MsV) (**c**) and the mean diameter of MsV (**d**) in the nonablation and mLSC ablation larvae at 7 dpf/1 dpt. 16 fish were observed in three independent experiments in each group. **e**, **f** Dorsal

confocal images of blood vessels and TUNEL signals on the *Tg(epd:mCherry-NTR; kdrl:GFP)* larval brains at 6 dpf/0 dpt. **e**, *n* = 22; **f**, *n* = 20. Each experiment was repeated three times independently with similar results. **g**, **h** Dorsal confocal images of mLSCs and pericytes on *Tg(epd:mCherry-NTR; abcc9^{BAC}:Gal4ff; UAS:GFP)* larval brains at 7 dpf/1 dpt. **g^{I}**, **h^{I}** showed the midbrain. **g^{II}**, **h^{II}** showed the hindbrain. **g**, *n* = 45; **h**, *n* = 46. Each experiment was repeated three times independently with similar results. Error bars, mean ± SEM. Unpaired two-tailed Student's *t*-test. *P* values included in the graphs. Source data are provided as a Source Data file. The white dashed boxes outline the enlarged areas. Scale bars: 50 μm.

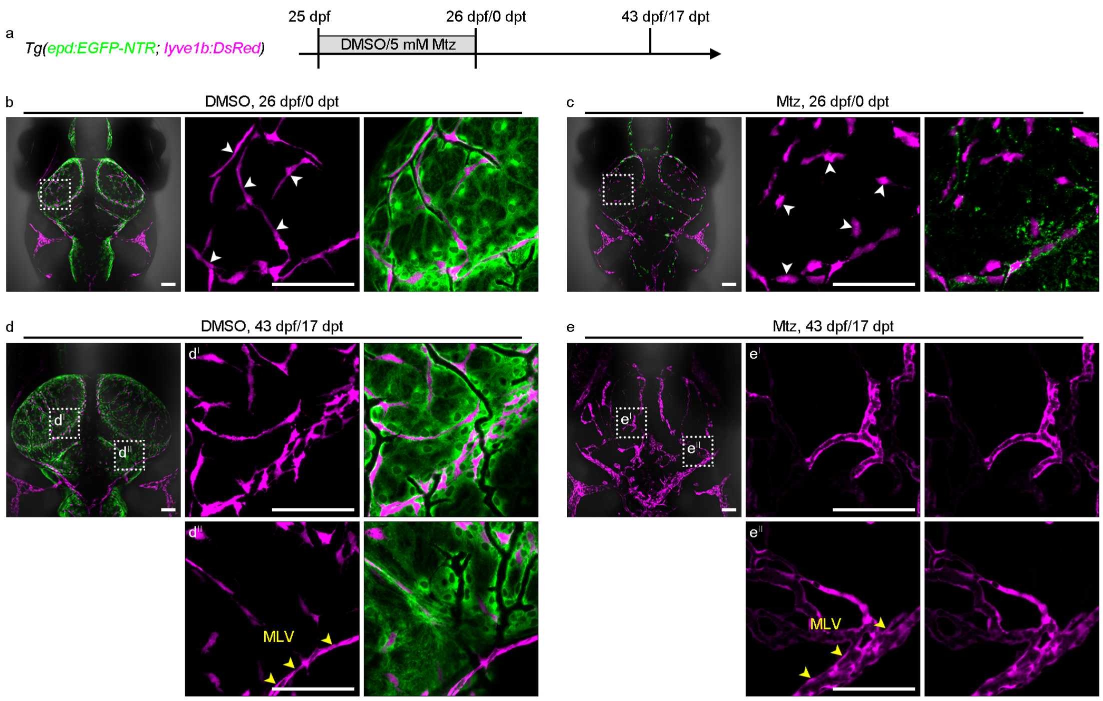

Supplementary Fig. 10 | The mLSCs are essential for maintaining the correct pattern of muLECs at the juvenile stage. a Schematic diagram showing experimental design for meningeal lymphatic supporting cell (mLSC) ablation. **b**, **d** Dorsal confocal images of mLSCs and mural lymphatic endothelial cells (muLECs) in DMSO-treated brains at 26 dpf/0 dpt (**b**, *n* = 40) and 43 dpf/17 dpt (**d**, *n* = 30). White arrowheads indicate muLECs. Yellow arrowheads indicate meningeal lymphatic vessels (MLV). **c** Dorsal confocal images of ablation of mLSCs and collapse of muLECs in the Mtz treated brain at 26 dpf/0 dpt. White arrowheads indicate collapsed muLECs. *n* = 37. The experiment was repeated three times independently with similar results. **e** Dorsal confocal images of muLEC-derived lymphatic vessels in Mtz-treated brains at 43 dpf/17 dpt. Yellow arrowheads indicate MLV. *n* = 26. The experiment was repeated three times

independently with similar results. The white dashed boxes outline the enlarged areas. Scale bars: 100 μm.

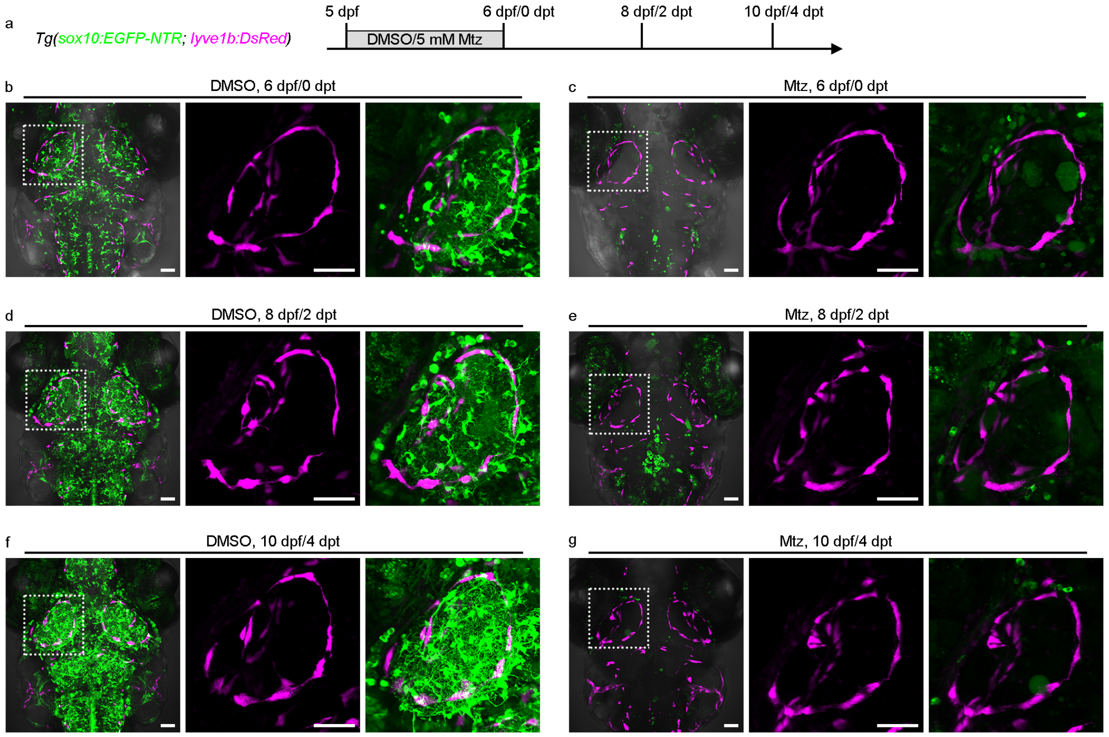

Supplementary Fig. 11 | Ablation of oligodendrocytes and oligodendrocyte progenitor cells does not induce morphological alterations in muLECs. a Schematic diagram showing experimental design for *sox10*-positive cell ablation**. b**, **d**, **f** Dorsal confocal images of mural lymphatic endothelial cells (muLECs) and *sox10*-positive cells in DMSO-treated larval brains at 6 dpf/0 dpt (**b**, *n* = 45), 8 dpf/2 dpt (**d**, *n* = 43) and 10 dpf/4 dpt (**f**, *n* = 40). **c**, **e**, **g** Dorsal confocal images of muLECs after ablation of *sox10*-positive cells in Mtz-treated larval brains at 6 dpf/0 dpt (**c**, *n* = 42), 8 dpf/2 dpt (**e**, *n* = 38) and 10 dpf/4 dpt (**g**, *n* = 37). Each experiment was repeated three times independently with similar results. The white dashed boxes outline the enlarged areas. Scale bars: 50 μm.

Supplementary Table 1. Primers used in this study

Name	Forward primer (5’-3’)	Reverse primer (5’-3’)
Primers used for *epd* and *sox10* promoter (Digestion and protection sites are underlined)		
epd	<u>CCGAAAGGGCCC</u>GTTGAATAATGTGCCCCAGG	<u>GGACCCACCGGT</u>CGTTGCTCTTTAACTTTCTTCAGG
sox10	<u>CCGAAAGGGCCC</u>GCTTTCCCGTGTACATCCAC	<u>GGACCCACCGGT</u>CGGTCCACTCGTTCTGCGGCC
Primers used for whole-mount in situ hybridization (WISH) and fluorescence in situ hybridization (FISH)-antibody staining (T7 promoter sequences are underlined)		
epd	GGCTGGGCTTCTCATCACTC	<u>TAATACGACTCACTATAGG</u>GTCGTGGAACAGGTCGAAGAA
ggctb	GATCCTCAGCTTCACGCTCG	<u>TAATACGACTCACTATAGG</u>CACATTCCTCCTGATCTGCGT
slc13a4	CGGATGGACGTCGCTTTTTG	<u>TAATACGACTCACTATAGG</u>GTGCAAAGCATCATGGACGG
apof	GCAAATCACAGCACCACCTAC	<u>TAATACGACTCACTATAGG</u>GATTCTCGTCATTGCTAGTTGGC
nid1b	GGACAGAGCAGCGTACTCAT	<u>TAATACGACTCACTATAGG</u>GCGCAAAACACTGCTATTGG
slc7a2	CCAAACTGAGTAAGCGCCAG	<u>TAATACGACTCACTATAGG</u>CTGGGGAACGCTGTGAATAAC
soul5	TGCACACGTTTCCTCTCTGC	<u>TAATACGACTCACTATAGG</u>GGGTAATGCACTGCTGTTCT
igfbp2a	CGCTCAGAAATGGTGTTCCG	<u>TAATACGACTCACTATAGG</u>GGGCAAGAGGGTAAGGGATG

Supplementary Table 2. Antibodies used in this study

Name	Company	Cat#	Dilution
Goat anti-GFP	Abcam	ab6658	1:2000
Mouse anti-mCherry	Abcam	ab125096	1:2000
Mouse anti-DsRed	Santa Cruz	SC-101526	1:2000
Rabbit Anti-Collagen I	Abcam	ab23730	1:1000
Anti-digoxigenin POD, Fab fragment	Roche	11207733910	1:2000
Anti-digoxigenin AP, Fab fragment	Roche	11093274910	1:2000
Donkey anti-goat IgG Alexa fluor 488-conjugated	Invitrogen	A11055	1:2000
Donkey anti-mouse IgG Alexa fluor 568-conjugated	Invitrogen	A10037	1:2000
Donkey anti-rabbit IgG Alexa fluor 568-conjugated	Invitrogen	A10042	1:2000
Donkey anti-mouse IgG Alexa fluor 647-conjugated	Invitrogen	A31571	1:2000
