# Supplementary material for: Meningeal lymphatic supporting cells govern the formation and maintenance of zebrafish mural lymphatic endothelial cells
Source: Nat Commun. 2024 Jul 2;15:5547. doi: 10.1038/s41467-024-49818-5 (PMC11220022; doi:10.1038/s41467-024-49818-5)
Supplement: Supplementary file 3 — Description of Additional Supplementary Files [file 41467_2024_49818_MOESM3_ESM.pdf]

Description of Additional Supplementary Files

File Name: Supplementary Data 1
Description: Top 200 marker genes in different meningeal cell clusters from Farnsworth et al.[34]. Related to Supplementary Fig. 3.

File Name: Supplementary Data 2
Description: FPKM expression level of all detected genes in the *epd*-positive cells and whole fish at 55 hpf and 5 dpf. Related to Fig. 3.

File Name: Supplementary Data 3
Description: Comparison of gene expression between the *epd*-positive cells and whole fish at 55 hpf. Related to Supplementary Fig. 5.

File Name: Supplementary Data 4
Description: Comparison of gene expression between the *epd*-positive cells and whole fish 5 dpf. Related to Supplementary Fig. 5.

File Name: Supplementary Movie 1
Description: **The appearance and increase of *epd*:EGFP-positive cells.** At approximately 18 somite stage (ss), the *epd*-positive cells first appear in the midbrain and subsequently increase in number. The double *Tg(epd:EGFP; kdrl:mCherry-Ras)* transgenic lines at 18-24 ss was used for the time-lapse imaging (*n* = 3). Duration of imaging is 180 minutes (1 frame/20 min). Scale bar, 200 μm.

File Name: Supplementary Movie 2
Description: **Migration trajectory of muLECs during muLEC sprouting.** The *epd*-positive cells are clustered early around the tracks where mural lymphatic endothelial cells (muLECs) about to migrate along, then muLECs migrate along the tracks provided by *epd*-positive cells. The double *Tg(epd:mCherry-NTR; lyve1b:EGFP)* transgenic lines at 60-106 hpf was used for the time-lapse imaging (*n* = 3). Three different colored arrowheads represent the migration paths of muLECs in different regions. Duration of imaging is 46.67 hours (1 frame/20 min). Scale bar, 200 μm.

File Name: Supplementary Movie 3
Description: **Changes of *epd*-positive cells and muLECs in *Tg(epd:mCherry-NTR; lyve1b:EGFP)* at 60-106 hpf during and after Mtz treatment.** The *epd*-positive cells gradually ablated and few mural lymphatic endothelial cells (muLECs) were able to migrate out of the optic choroidal vascular plexus (OCVP) accompanied by Mtz treatment. The transgenic lines with Mtz treatment from 48 hpf to 72 hpf was used for the time-lapse imaging (*n* = 3). Duration of imaging is 46.33 hours (1 frame/20 min). Scale bar, 200 μm.

File Name: Supplementary Movie 4
Description: **Changes of mLSCs and muLECs in *Tg(epd:EGFP-NTR; lyve1b:DsRed)* at 5 dpf to 6.5 dpf during and after Mtz treatment.** The meningeal lymphatic supporting cells (mLSCs) gradually ablated and the elongated mural lymphatic endothelial cells (muLECs)

progressively collapsed into round shape accompanied by Mtz treatment. The larvae with Mtz treatment from 5 dpf to 6 dpf was used for the time-lapse imaging (n = 3). Duration of imaging is 33 hours (1 frame/20 min). Scale bar, 200 µm.
