# Supplementary material for: Meningeal lymphatic supporting cells govern the formation and maintenance of zebrafish mural lymphatic endothelial cells
Source: Nat Commun. 2024 Jul 2;15:5547. doi: 10.1038/s41467-024-49818-5 (PMC11220022; doi:10.1038/s41467-024-49818-5)
Supplement: Supplementary file 12 — Reporting Summary [file 41467_2024_49818_MOESM12_ESM.pdf]

nature portfolio

Corresponding author(s): Lingfei Luo
Last updated by author(s): Jun 7, 2024

Reporting Summary

Nature Portfolio wishes to improve the reproducibility of the work that we publish. This form provides structure for consistency and transparency in reporting. For further information on Nature Portfolio policies, see our Editorial Policies and the Editorial Policy Checklist.

Statistics

For all statistical analyses, confirm that the following items are present in the figure legend, table legend, main text, or Methods section.

n/a	Confirmed	
☐	☒	The exact sample size (*n*) for each experimental group/condition, given as a discrete number and unit of measurement
☐	☒	A statement on whether measurements were taken from distinct samples or whether the same sample was measured repeatedly
☐	☒	The statistical test(s) used AND whether they are one- or two-sided<br>*Only common tests should be described solely by name; describe more complex techniques in the Methods section.*
☒	☐	A description of all covariates tested
☒	☐	A description of any assumptions or corrections, such as tests of normality and adjustment for multiple comparisons
☐	☒	A full description of the statistical parameters including central tendency (e.g. means) or other basic estimates (e.g. regression coefficient) AND variation (e.g. standard deviation) or associated estimates of uncertainty (e.g. confidence intervals)
☐	☒	For null hypothesis testing, the test statistic (e.g. *F*, *t*, *r*) with confidence intervals, effect sizes, degrees of freedom and *P* value noted<br>*Give P values as exact values whenever suitable.*
☒	☐	For Bayesian analysis, information on the choice of priors and Markov chain Monte Carlo settings
☒	☐	For hierarchical and complex designs, identification of the appropriate level for tests and full reporting of outcomes
☒	☐	Estimates of effect sizes (e.g. Cohen's *d*, Pearson's *r*), indicating how they were calculated

Our web collection on statistics for biologists contains articles on many of the points above.

Software and code

Policy information about availability of computer code

Data collection

Zeiss imaging software (2.6) and ZEISS LSM780 and LSM880 were used for confocal imaging.
FlowJo software (10.5.3) of the Beckman Coulter Moflo XDP cell sorter was used for cell sorting.
Zeiss Lightsheet Z.1 and ZEISS LSM 880 were used for time-lapse imaging.
ZEISS SteREO Discovery.V20 was used for imaging whole-mount in situ hybridizated sample.

Data analysis

Quantifications were performed using GraphPad Prism 8.0.1.
The flow cytometry results were analyzed using FlowJo software (10.5.3).
The heatmaps in Fig. 3e, f were created using Heatmap Illustrator (HemI) version 1.0.3.7.
All movies were handled with Fiji (ImageJ) 2.14.0 and ZEN2010 Imaging software.
Bulk RNA sequencing data analysis: The raw data aligned to the zebrafish (Danio rerio) reference genome (GRCz11.96). Clean Reads are obtained by processes such as removing Raw Reads from low-quality sequences to complete data processing, and all subsequent analysis is based on Clean Reads.
Single-cell RNA sequencing data analysis: ScRNAseq data were preprocessed and normalized using the R package "Seurat v4.3.0". The epd-positive cells, fibroblasts, mural cells, neural progenitor cells, immune cells, and endothelial cells were picked out for further analysis according to the classical marker genes of each cell type. The Uniform Manifold Approximation and Projection (UMAP) plot was obtained by dimensionality reduction analysis using the RunUMAP function (R package "Seurat v4.3.0"). The FindAllMarkers function (R package "Seurat" v4.3.0) was used to identify the highly expressed genes in each cluster, and the genes with padj < 0.05 were taken to be sorted by avg_log2FC, and the genes in the top 50 avg_log2FC were taken to be plotted by heatmap (R package "pheatmap 1.0.12"), take the genes in the top 20 of avg_log2FC of epd-positive cell population and plot them by dotplot (R package "Seurat v4.3.0"). Violin plot of each cell-specific marker gene using the VlnPlot function in the R package "Seurat v4.3.0". For Principle component analysis (PCA), the epd-positive cells in scRNAseq data were first split into 3 groups for pseudo-bulk process according to the mean value of expression, then ComBat_seq in R package "sva 3.42.0"

was used to eliminate the batch effect on bulk RNA-sequencing data (epd-positive cells at 55 hpf and 5 dpf, and whole zebrafish at 55 hpf and 5 dpf) generated in this study and the epd-positive cells in single-cell RNA-sequencing data that were split into 3 groups, and finally PCA clustering analysis was performed with R package "ape 5.7.1".

For manuscripts utilizing custom algorithms or software that are central to the research but not yet described in published literature, software must be made available to editors and reviewers. We strongly encourage code deposition in a community repository (e.g. GitHub). See the Nature Portfolio guidelines for submitting code & software for further information.

Data

Policy information about availability of data

All manuscripts must include a data availability statement. This statement should provide the following information, where applicable:

- Accession codes, unique identifiers, or web links for publicly available datasets
- A description of any restrictions on data availability
- For clinical datasets or third party data, please ensure that the statement adheres to our policy

The RNA sequencing data generated in this paper have been deposited in the Genome Sequence Archive in National Genomics Data Center under the accession code GSA: CRA016778 [https://ngdc.cncb.ac.cn/gsa/search?searchTerm=CRA016778]. Previous published scRNA-seq data that were re-analyzed in this study are available in the National Centre for Biotechnology (NCBI) SRA: PRJNA564810 [https://www.ncbi.nlm.nih.gov/sra/?term=PRJNA564810]34. All other relevant data that support the findings of this study are available within the article, its Supplementary Information and its Supplementary Data, or from the corresponding author upon reasonable request.

Research involving human participants, their data, or biological material

Policy information about studies with human participants or human data. See also policy information about sex, gender (identity/presentation), and sexual orientation and race, ethnicity and racism.

Reporting on sex and gender	N/A
Reporting on race, ethnicity, or other socially relevant groupings	N/A
Population characteristics	N/A
Recruitment	N/A
Ethics oversight	N/A

Note that full information on the approval of the study protocol must also be provided in the manuscript.

Field-specific reporting

Please select the one below that is the best fit for your research. If you are not sure, read the appropriate sections before making your selection.

☒ Life sciences ☐ Behavioural & social sciences ☐ Ecological, evolutionary & environmental sciences

For a reference copy of the document with all sections, see nature.com/documents/nr-reporting-summary-flat.pdf

Life sciences study design

All studies must disclose on these points even when the disclosure is negative.

Sample size	Sample sizes chosen was based on recognised zebrafish standards in the field and previously published literature (e.g. PMID: 28459441, 37311876 and 28395729). We have described the exact sample size for each experiment in the manuscript. No statistical methods were used to predetermine the sample size.
Data exclusions	No data were excluded from analyses.
Replication	With the exception of the RNA sequencing experiments, all experiments were verified with at least 3 biological replicates and/or independent experiments. The exact number of repetitions for all experiments is illustrated in the legend, and our attempts at repetition were successful.
Randomization	All experiments that compared treatment groups were conducted using randomly assigned embryos, larvae, juveniles, and adults.
Blinding	As different transgenic zebrafish embryos, larvae, juveniles, and adults were used in the experiments, researchers were not blinded to group assignment during data collection and/or analysis.

Reporting for specific materials, systems and methods

We require information from authors about some types of materials, experimental systems and methods used in many studies. Here, indicate whether each material, system or method listed is relevant to your study. If you are not sure if a list item applies to your research, read the appropriate section before selecting a response.

Materials & experimental systems

n/a	Involved in the study
☐	☒ Antibodies
☒	☐ Eukaryotic cell lines
☒	☐ Palaeontology and archaeology
☐	☒ Animals and other organisms
☒	☐ Clinical data
☒	☐ Dual use research of concern
☒	☐ Plants

Methods

n/a	Involved in the study
☒	☐ ChIP-seq
☐	☒ Flow cytometry
☒	☐ MRI-based neuroimaging

Antibodies

Antibodies used	Primary antibodies used were Anti-digoxigenin AP, Fab fragment (1:2000, 11093274910, Roche), Anti-digoxigenin POD, Fab fragment (1:2000, 11207733910, Roche), anti-GFP (1:2000, ab6658, Abcam), anti-mCherry (1:2000, ab125096, Abcam), anti-DsRed2 (1:2000, sc-101526, Santa Cruz), Anti-Collagen I (1:1000, ab23730, Abcam). Secondary antibodies used were Donkey anti-goat IgG Alexa fluor 488-conjugated (1:2000, A11055, Invitrogen), Donkey anti-mouse IgG Alexa fluor 568-conjugated (1:2000, A10037, Invitrogen), and Donkey anti-rabbit IgG Alexa fluor 568-conjugated (1:2000, A10042, Invitrogen).
Validation	All antibodies used in this study were commercially sourced.<br>Anti-digoxigenin AP, Fab fragment, https://www.sigmaaldrich.cn/product/roche/11093274910.<br>Manufacturer: The conjugate can be used for the detection of digoxigenin-labeled compounds.<br>Reference: PMID:21356874.<br><br>Anti-digoxigenin POD, Fab fragment, https://www.sigmaaldrich.cn/product/roche/11207733910.<br>Manufacturer: The conjugates can be used for the detection of digoxigenin-labeled compounds.<br>Reference: PMID:32366356.<br><br>anti-GFP, https://www.abcam.com/products/primary-antibodies/biotin-gfp-antibody-ab6658.html.<br>Manufacturer: Designed to detect GFP and its variants in ELISA (sandwich or capture), immunoblotting and immunoprecipitation.<br>Reference: PMID:35693809.<br><br>anti-mCherry, https://www.abcam.com/products/primary-antibodies/mcherry-antibody-1c51-ab125096.html.<br>Manufacturer: Recombinant full length protein corresponding to mCherry.<br>Reference: PMID:36198703<br><br>anti-DsRed2, https://www.scbt.com/p/dsred2-antibody-25.<br>Manufacturer: DsRed2 exhibits high signal to noise ratio and distinct spectral properties, making it a useful fusion tag for various proteins.<br>Reference: PMID:16841215.<br><br>Anti-Collagen I, https://www.abcam.com/products/primary-antibodies/collagen-i-antibody-ab23730.html.<br>Manufacturer: Full length native protein (purified) corresponding to Collagen I. Purified collagen type I from tuna fish skin.

Animals and other research organisms

Policy information about studies involving animals; ARRIVE guidelines recommended for reporting animal research, and Sex and Gender in Research

Laboratory animals	Wild-type and transgenic zebrafish and mutant zebrafish used in this study were from the AB strain. The following transgenic and mutant lines were used: Tg(epd:EGFP)cq188, Tg(epd:EGFP-NTR)cq189, Tg(epd:mCherry-Ras)cq190, Tg(epd:mCherry-NTR)cq191, Tg(epd:H2B-GFP)cq192, Tg(epd:H2B-mCherry)cq193, Tg(lyve1b:EGFP-NTR)cq194, and Tg(sox10:EGFP-NTR)cq195. The previously published zebrafish lines used were Tg(lyve1b:DsRed)cq27, Tg(lyve1b:EGFP)cq86, Tg(abcc9BAC:Gal4ff)ncv34, Tg(pdgfrbBAC:GFP)ncv22, Tg(acta2:GFP)ca7, Tg(coro1a:Kaede)cq22, Tg(lyz:GFP)nz117, Tg(mpeg1:GFP)gl22, Tg(prox1aBAC:KalTA4; UAS:TagRFP)nim5, Tg(nkx2.2a:GFP)ia3, Tg(elavl3:GFP) knu3, Tg(kdrl:mCherry-Ras)s896, Tg(fli1:GFP)y1, Tg(kdrl:GFP)s843, Tg(fli1:nEGFP)y7, and casper. Age of 18 somite stage, 24 hpf, 48 hpf, 55 hpf, 3 dpf, 4 dpf, 5 dpf, 6 dpf, 7 dpf, 8 dpf, 10 dpf, 11 dpf, 14 dpf, 17 dpf, 20 dpf, 25 dpf, 26 dpf, 43 dpf, 4 mpf and 6 mpf and length of 7 mm zebrafish were used for experiments.
Wild animals	No wild animals involved in this study.
Reporting on sex	All zebrafish were used with equal number of males and females when sex determination has occurred.
Field-collected samples	This study did not include samples collected from the field.
Ethics oversight	All animal experiments were approved by the Institutional Animal Care and Use Committee (IACUC) of Southwest University

Ethics oversight	Laboratory Animal Center. All animal procedures followed standard conditions in accordance with the regulations of the Ethics Committee of Southwest University (Chongqing, China).

Note that full information on the approval of the study protocol must also be provided in the manuscript.

Flow Cytometry

Plots

Confirm that:

☒ The axis labels state the marker and fluorochrome used (e.g. CD4-FITC).

☒ The axis scales are clearly visible. Include numbers along axes only for bottom left plot of group (a 'group' is an analysis of identical markers).

☒ All plots are contour plots with outliers or pseudocolor plots.

☒ A numerical value for number of cells or percentage (with statistics) is provided.

Methodology

Sample preparation	Heads of transgenic Tg(epd:EGFP-NTR; lyve1b:DsRed) zebrafish at 55 hpf and 5 dpf were separately dissected and placed in 1 mL 1X PBS on ice. They were then washed with 1 mL 1X PBS once and centrifuged at 4000 rcf at 4 °C for 2 minutes. Next, the supernatant was removed and heads were dissociated with a mixture solution of 200 µL PBS-EDTA (1 mM EDTA in 1X PBS) and 50 µL 2.5% trypsin. The homogenized cell suspension was centrifuged at 4000 rcf at 4 °C for 2 minutes before being washed with 1 mL 1X PBS twice. The cells were then resuspended in 200 µL 1X PBS within 5 minutes and the cell suspension was collected by filtering through a 40 µm cell strainer into 2 mL EP tube. Wild-type zebrafish heads at 55 hpf and 5 dpf were operated using the same dissociation procedure as a fluorescent-negative control for FAC sorting. Subsequently, cell sorting was performed using a flow cytometry (Moflo XDP, Beckman) to obtain 55 hpf and 5 dpf of EGFP-NTR (+) DsRed (-) cells as two biological replicates.
Instrument	Beckman Coulter Moflo XDP cell sorter.
Software	Cell sorting was performed using FlowJo software (10.5.3) of the Beckman Coulter Moflo XDP cell sorter.
Cell population abundance	About 100 epd-EGFP-NTR-positive cells per replicate at 55 hpf and 5 dpf were collected using Moflo XDP cell sorter and used for RNA sequencing.
Gating strategy	Gating strategy illustrated in Fig. 3c, d.

☐ Tick this box to confirm that a figure exemplifying the gating strategy is provided in the Supplementary Information.
